# Concentrations and Geographical Variations of Selected Toxic Elements in Meat from Semi-Domesticated Reindeer (*Rangifer tarandus tarandus L.*) in Mid- and Northern Norway: Evaluation of Risk Assessment

**DOI:** 10.3390/ijerph9051699

**Published:** 2012-05-04

**Authors:** Ammar Ali Hassan, Magritt Brustad, Torkjel M. Sandanger

**Affiliations:** 1 Centre for Sami Health Research, Department of Community Medicine, Faculty of Health Sciences, University of Tromsø, N-9037 Tromsø, Norway; Email: magritt.brustad@uit.no (M.B.); torkjel.sandanger@uit.no (T.M.S.); 2 Norwegian Institute for Air Research (NILU), Fram Centre, N-9296 Tromsø, Norway; Email: tsa@nilu.no

**Keywords:** reindeer meat, toxic elements, Norway, Arctic food, risk assessment

## Abstract

Meat samples (n = 100) from semi-domesticated reindeer (*Rangifer tarandus tarandus L.*) were randomly collected from 10 grazing districts distributed over four Norwegian counties in 2008 and 2009. The main aim was to study concentrations and geographical variations in selected toxic elements; cadmium (Cd), lead (Pb), arsenic (As), copper (Cu), nickel (Ni) and vanadium (V) in order to assess the risk associated with reindeer meat consumption. Sample solutions were analysed using an inductively coupled plasma high resolution mass spectrometer (ICP-HRMS), whereas analysis of variance (ANOVA) was used for statistical analyses. Geographical variations in element concentrations were revealed, with As and Cd demonstrating the largest geographical differences. No clear geographical gradient was observed except for the east-west downward gradient for As. The As concentrations were highest in the vicinity of the Russian border, and only Cd was shown to increase with age (*p* < 0.05). Sex had no significant effect on the concentration of the studied elements. The concentrations of all the studied elements in reindeer meat were generally low and considerably below the maximum levels (ML) available for toxic elements set by the European Commission (EC). Thus, reindeer meat is not likely to be a significant contributor to the human body burden of toxic elements.

## 1. Introduction

In recent years, there has been considerable concern over the extent of toxic elements in the environment and their possible negative health effects. The limited amounts of data on local sources, as well as the increased number of slaughtered reindeer, necessitate a need for the continuous monitoring of such elements in meat to secure food safety for the consumer [1,2,3,4,5,6]. The concentration of toxic elements in animal tissues depends on the animal’s species, dietary concentration of the element, tissue absorption, concentrations of other elements in the animal tissue and the body’s homeostatic control mechanism for the element [7]. These elements are toxic for both human and animals, and cause a range of diseases [8,9,10,11,12]. Furthermore, the highest concentrations have been found in tissues such as kidneys, liver and bones [1,3,13,14]. Geographical variations in the concentrations of toxic elements in meat, liver and kidneys from reindeer have previously been demonstrated [15,16,17].

Both natural and anthropogenic components contribute to geographical variations in the concentration of toxic elements. Moreover, the difference in exposure patterns may be due to different type of diets, as both animal diet preferences and the type of vegetation, vary from one place to another. Differences in exposure are therefore expected in areas with a different animal density and a different availability of lichens. Toxic metal concentrations in animals have been reported to be associated with the distance to pollution sources, thus districts located close to the sources have higher concentrations than other areas [18,19]. Wind frequency and direction also influence the atmospheric deposition of toxic metals [20].

The main reindeer summer/autumn feed are grasses, sedges, twigs, leaves and mushrooms [21]. Some plants (metallophytes) have the ability to absorb and accumulate more toxic elements from the soil in their tissues, even when soil concentrations are low, compared with other ones within the same geographical area. Additionally, the elements composition of plants vary within species as well as at the various stages of plant growth [22]. The decreased pH (increased acidity) of the soil as a result of acid rain affects the solubility and mobility of some toxic elements (e.g., an increase in the case of Cd) [23,24]. In this way, their uptake by plants and accumulation by animals may increase. However, areas in the southernmost part of Norway are the ones most affected by acid rain due to the long-range atmospheric transportation from Central and Western Europe compared to areas in the north of Norway [25,26]. As a consequence of this, Norwegian cervine animals—particularly reindeer—from southern Norway have previously exhibited elevated liver and kidney cadmium levels [1].

Lichens are the main reindeer winter feed, with a varied distribution among the different grazing districts, and have the ability to accumulate toxic elements from the atmosphere [27,28,29]. In former studies, lichens also revealed the greatest variation in metal concentrations compared with other plants collected from contaminated and reference areas in Swedish Lapland [19].

Areas close to the Norwegian-Russian border are the primary issue of concern due to the location of the two Russian towns of Nikel (nickel smeltery) and Zapoljarny (briquette industry). The town of Nikel is located 7 km from the Norwegian border, while the town of Zapoljarny is located 30 km further east. The high atmospheric level of Ni, Cu, Co and As previously measured from the Norwegian area of Svanvik close to the Russian border was reported to be due to the release from the smelting activities in Nikel and Zapoljarny [20,30]. In addition to the known Russian sources, the presence of local mining facilities and military activities in some districts acts as potential point sources, and has been an issue of concern.

The main purpose of this project was to study the concentrations and geographical variations of selected toxic elements in meat from semi-domesticated reindeer in the selected grazing districts in mid- and northern Norway in order to assess the risk associated with reindeer meat consumption. 

## 2. Materials and Methods

### 2.1. Sample Collection and Preparation

**Figure 1:**
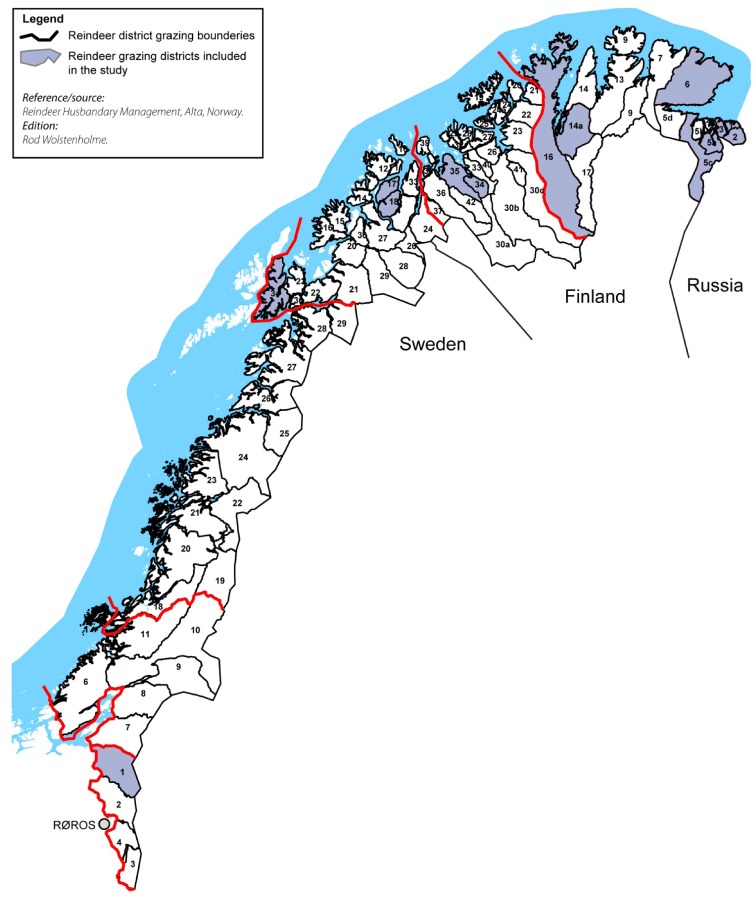
Map of the study area.

Meat samples (n = 100) from the neck-region were randomly collected from semi-domesticated reindeer in 10 different grazing districts located in northern and middle Norway in the period from October–December 2008 and September–December 2009. The samples were collected from four different counties distributed as follows: Finnmark County (seven districts), Troms County (one district), Nordland County (one district) and South-Trøndelag County (one district). The selection of the 10 districts was based on obtaining a broad geographical range and the susceptibility of certain districts to pollution from mines, smeltery and military activities (Figure 1). The selection of seven districts from Finnmark County which is the biggest and northernmost Norwegian county was based on the fact that this county has the largest number of semi-domesticated reindeer and 50% of the total number of reindeer grazing districts in Norway. The districts of Eastern Sør-Varanger, Pasvik/Sør-Varanger and Varanger Peninsula are located near the Norwegian-Russian border, where the contamination from mines and smeltery activities is taking place. Spierttagáisá is located in a military activity area, whereas Karasjok West is a neighbouring district. There is also mining work taking place in Ábborašša, which is close to Fávrrosorda a neighbouring district.

Our focus was on young animals (1.5 years old), which represented 77% of the total samples. However, calves (approximately10 months old) and older animals (>2 years old) with the respective proportions of 12% and 11% had to be selected due to the limited availability of 1.5 year olds in some districts (n = 4). There were 52 males and 48 females of the 100 selected reindeer.

All samples were collected directly after the slaughter/dressing process and carcass weighing in acid-rinsed glasses. The glasses were labelled with sample type, carcass number, district name/number and date of sample collection. The samples were kept cool in a cooling box (at approximately 4 °C) and then moved the same day to a −20 °C freezer until they were shipped frozen to the laboratory for analysis. All of the animals from the collected samples were healthy, *i.e*., had passed the veterinary meat inspection.

### 2.3. Chemical Analysis

The samples were separately decomposed using a microwave oven technique, with concentrated supra-pure HNO_3_ (5 mL) and H_2_O_2_ (3 mL) being added to the decomposed sample (0.6–0.7 g) before undergoing microwave oven treatment. The following temperature regimes were subsequently used in the microwave oven: 20–50 °C (5 min), 50–100 °C (10 min), 100–180 °C (5 min) and 180 °C (15 min). After cooling the heated decomposed sample, the solution was diluted to 50 ml. The sample solution was then analysed using an inductively coupled plasma high resolution mass spectrometer (ICP-HRMS), Bremen, Germany. All standards and calibration solutions contained 1 ppb Rhenium (Re) as the internal standard, together with 1% nitric acid (HNO_3_). The calibration curve was verified by standard quality control (QC) sample, National Institute of Standards and Technology (NIST, USA). The resolutions used for the elements were low (at 10) for Cd and Pb, middle (at 20) for Cu, V and Ni, and high (at 30) for As. The lens adjustment was optimized daily for maximum intensity and top separation. The analyses were done by the NILU (Norwegian Institute for Air Research) Laboratory (Kjeller, Norway). The laboratory is accredited for the methods used in the analyses according to NS-EN ISO/IEC 17025, No. TEST008. The limits of detections (LODs) for the studied toxic elements were three times standard deviation (SD) of the laboratory blanks, whereas the limits of quantifications (LOQs) were 10 times the SD of the blanks, decomposed simultaneously with the meat samples.

### 2.4. Statistical Analysis

STATA/SE 11.0 for Windows (STATA Corp. College Station, TX, USA) was used for the statistical analyses. Laboratory results for elements below the limit of detection (LOD) were given a numeric value at half the LOD (LOD/2) according to accepted statistical practice [31]. All toxic element concentrations, except for Cu which was normally distributed, were positively skewed (skewed to the right). Therefore, all concentrations were log transformed to obtain a normal distribution before statistical evaluation. A standardized residuals test was conducted prior to the log transformation in order to check for possible outliers (observations that were more than three standard deviations from the mean). Consequently, all outliers were removed (n = 10, details in results and discussion).

An analysis of variance and covariance (ANOVA), with the specific element as a dependent (outcome) variable and the district, age and sex as independent (explanatory) variables, was used to test for the effect of the independent variables on toxic element concentrations. Bonferroni multiple comparison tests were used to test for significant differences in toxic element concentrations among the 10 grazing districts, using the specific element as an outcome and the districts as a factor variable. A Welch test was used whenever the homogeneity of variance was violated. The level of statistical significance was set at *p* < 0.05 for all the statistical analyses. 

## 3. Results and Discussion

The present study is unique since it is the first of its kind to include such a large number of animals to study the concentrations and geographical variability of toxic elements in meat from semi-domesticated reindeer. Moreover, the assessment was done using meat that is more relevant for human consumption. Further, the reindeer originated from 10 different grazing districts covering a large geographical area extending from the middle to the northernmost part of Norway.

The overall concentrations of toxic elements in meat samples from all animals (n = 100) are presented in Table 1. The results were presented as percentage (%) of samples above LOD, geometric mean (GM), arithmetic mean ± standard deviation (AM ± SD), range (Min-Max) and coefficient of variation (CV%). Geographical variations in toxic element concentrations between some districts were demonstrated (Table 2). Detailed result of the multiple comparison tests for concentrations among the 10 grazing districts are presented as supplementary material (Table 1S). Sex had not any significant influence on toxic element concentrations.

**Table 1:** The overall toxic element concentrations (ng/g ww) in reindeer meat.

Element	n	% detected	GM	AM ± SD	Min.	Max.
Cd	98	99	1.7	2.5 ± 2.5	<0.37	11
Pb	98	92	1.5	2.7 ± 3.1	<0.19	20
As	99	100	8.1	20 ± 32.1	0.7	135
Cu	98	100	1,439	1,469 ± 296	830	2,357

**Legend:** n = Number of observations. % detected= Percentage of samples within the limit of detection (LOD); GM = Geometric mean. AM ± SD = Arithmetic mean ± standard deviation. Min-Max= Minimum–Maximum; CV = Coefficient of variation. **Note:** The limits of detection (LODs) for the toxic elements (e.g., 0.37 and 0.19) were stipulated by calculating the mean value of all LODs for the specific element. Numbers of samples were less than 100 due to removal of outliers.

Toxic element concentrations in 10 samples were outliers and have been removed from the statistical analyses. The detected outliers were: One animal (1.5 years) from Eastern Sør-Varanger with an As concentration of 161 ng/g ww; two animals from Kanstadfjord; a 1.5 years old and an older one (>2 years) with Cd concentrations of 13 and 15 ng/g ww, respectively; two animals: a calf from Varanger Peninsula and an older one (>2 years) from Spierttagáisá with Pb concentrations of 28 and 23 ng/g ww, respectively; two animals (1.5 years) from Fávrrosorda and Karasjok West with Cu concentrations of 161 and 2837 ng/g ww, respectively; one animal (1.5 years) from Tromsdalen with Ni concentration of 19 ng/g ww; two animals (1.5 years) from Spierttagáisá and Karasjok West with V concentrations of 5.1 and 8.3 ng/g ww, respectively.

### 3.1. Cadmium (Cd)

Cadmium was detected in 99% of the meat samples, and had the second largest geographical variation after arsenic. The geographical variations in cadmium concentrations are presented in Table 2 and Table 3. 

The presence of older animals in some districts revealed a significant district × age interaction, therefore stratified data were given for Cd concentrations in districts with mixed age groups (Table 3). Calves and young animals demonstrated no significant difference in Cd concentration across geography (GM = 0.9 and 1.5, for calves and young animals respectively), while older animals (>2 years old) showed a higher Cd concentration (GM = 4.3, *p* < 0.01). The increase in Cd concentration with age was in accordance with previous studies [15,16,32]. A Finnish study on toxic metals in reindeer from four districts in Lapland has shown comparable muscle Cd concentrations ranging from 1 to 6 ng/g ww [15]. Moreover, Cd concentrations from the present study were up to 100 times lower than those found in reindeer muscle (mean = 580 ng/g ww) originating from Karelia in the Russian Federation [33].

Cd concentrations in some districts from the present study were higher than those from reindeer muscles collected from three Greenlandic districts, whereas concentrations from the districts of Essand/Røros, Ábborašša, Kanstadfjord and Fávrrosorda were comparable to that reported from one district (GM = 3 ng/g ww) [17]. The high Cd concentration in Kanstadfjord was due to presence of some older animals (n = 4). Furthermore, high Cd concentration in Fávrrosorda might be due to atmospheric deposition from the neighbouring district Ábborašša with its gold mining activity. The high concentration in Essand was probably a result of acid rain due to atmospheric transport of industrial pollution from Europe which has previously been reported to affect southern Norway to a greater extent than northern part of the country [34,35,36].

### 3.2. Lead (Pb)

Pb was detected in 92% of the meat samples. Concentrations of the Pb among the various grazing districts did not vary to the same degree as for As and Cd (Table 2). 

The study on Finnish reindeer by Rintala and colleagues had also reported little differences in concentrations of Pb among different Lapland areas [15]. Even so, the Pb concentrations detected in this study were much lower (10 to 20 times) than those reported in reindeer meat by the same reference above. The Pb concentration from Fávrrosorda in the present study (GM= 7.4, AM= 8.6 ng/g ww) was an exception and could be compared to the concentration from the southern, western and eastern parts of Finnish Lapland, in which the Pb concentration was half of that detected from northern Lapland [15]. The mean Pb concentration of 2.14 µg/g ww previously measured from the Karelian reindeer muscle in Russia was 289 times higher than the greatest level of GM= 7.4 ng/g ww measured in the present study [33].

The Pb concentration was five times higher (*p* < 0.01) in Fávrrosorda (a neighbouring district) compared to Ábborašša (gold mining facilities). This suggests that the deposition of lead occurs in the neighbourhood of the mining area rather than the mining area itself, which is based on the wind direction and is in accordance with results presented elsewhere [19]. The Pb concentration measured from Fávrrosorda in this study was in good agreement with those from unknown point sources in Greenlandic caribou and reindeer muscles from Isortoq (GM = 7 ng/g ww) in the north and Akia (GM = 6 ng/g ww) in the middle part of the country [17]. However, Pb concentrations in the remaining districts in the present study were two to four times lower than those reported by the Greenlandic study.

As for Cd, high Pb concentration in Fávrrosorda might be due to atmospheric deposition from the neighbouring district Ábborašša with its gold mining activity.

**Table 2:** Concentrations of toxic elements (ng/g wet wt) in reindeer meat (n = 90) from the ten different grazing districts.

District											
		ES-Varanger	Pasvik	Varanger P	Spierttagáisá	Karasjok	Ábborašša	Fávrrosorda	Tromsdalen	Kanstadfjord	Essand
**Cd**	GM	0.9	1.5	1.4	0.9	0.9	2.2	4.3	0.9	5.1	3.6
	AM ± SD	0.9 ± 0.7	1.9 ± 2	2.9 ± 3.5	0.9 ± 0.5	1.1 ± 0.6	2.9 ± 2.5	4.8 ± 1.9	0.9 ± 0.3	4.7 ± 2.9	4.4 ± 2.8
	Min-Max	0.5–2.8	0.8–7.6	0.4–10.4	0.4–2.1	0.6–2.6	0.9–8.2	1.5–7.4	<0.37–1.4	1.6–10.8	1.1–8.5
	CV%	69	103	121	53	54	85	41	31	63	64
	n	10	10	10	10	10	10	10	10	8	10
**Pb**	GM	0.4	1.9	1.6	1.2	1	1.6	7.4	0.9	1.7	2.7
	AM ± SD	0.7 ± 0.5	2.3 ± 1.9	2.1 ± 1.4	1.9 ± 2.1	2.2 ± 2.5	1.7 ± 1.6	8.6 ± 4.9	1.9 ± 2.5	2.5 ± 1.9	2.9 ± 1.2
	Min-Max	<0.23–1.5	1–7.8	0.3–4.4	<0.22–5.8	<0.12–8.3	0.18–4.6	3.1–20	<0.1–7.3	0.27–5.6	1.6–5.9
	CV%	81	84	68	111	114	96	58	128	79	42
	n	10	10	9	9	10	10	10	10	10	10
**As**	GM	106	48	10	5.9	8.8	6.9	1.9	1.5	3.9	7.2
	AM ± SD	107 ± 17	52 ± 19	11 ± 3.3	6.4 ± 2.5	8.9 ± 1.5	6.9 ± 1.2	2.2 ± 1.1	1.6 ± 1.8	4.1 ± 0.7	7.3 ± 1.7
	Min-Max	84–135	27–79	7–16	2.5–11	6.3–11	4.9–8.4	0.9–3.9	0.7–2.9	2.9–4.9	4.7–10
	CV%	16	38	30	39	17	17	49	48	17	24
	n	9	10	10	10	10	10	10	10	10	10
**Cu**	GM	1,700	1,700	1,300	1,400	1,500	1,600	1,200	1,300	1,300	1,600
	AM ± SD	1,700 ± 292	1,702 ± 296	1,326 ± 194	1,444 ± 188	1,549 ± 276	1,564 ± 231	1,208 ± 274	1,306 ± 167	1,287 ± 275	1,608 ± 295
	Min-Max	1,300–2,100	1,300–2,100	1,100–1,600	1,300–1,900	1,200–2,100	1,300–2,100	800–1,700	1,100–1,600	800–1,600	1,300–2,400
	CV%	17	17	15	13	18	15	23	13	21	18
	n	10	10	10	10	9	10	9	10	10	10
**Ni**	GM	<6.7	<7.2	0.8	<9.5	<11	<7.1	1.2	1.3	<5.7	<1.5
	AM±SD	<6.7	<7.2	1.2±1.1	<9.5	<11	<7.1	2.7±2.5	2.1±4.5	<5.7	<1.5
	Min.-Max	<6.7–<14	<7.2–<13	<0.4–3.6	<9.5–<39	<11–11	<7.1–<18	<0.1–7.1	<0.9–11	<5.7–<20	<1.5–5.8
	CV%			93				93	162		
	n	10	10	10	10	10	10	10	9	10	10
**V**	GM	<0.01	<0.02	<2.7	0.2	<0.26	<0.24	<1.9	<0.9	<0.09	<0.02
	AM ± SD	<0.01	<0.02	<2.7	0.3 ± 0.6	<0.26	<0.24	<1.9	<0.9	<0.09	<0.02
	Min-Max	<0.01–0.2	<0.02–0.3	<2.7–<3.8	<0.09–3.8	<0.02–8.3	<0.24–0.39	<1.9–<3.6	<0.9–3.5	<0.09–0.2	<0.02–<0.2
	CV%				197						
	n	10	10	10	9	9	10	10	10	10	10

**Legend:** GM = Geometric mean. AM = Arithmetic mean. SD = Standard deviation. Min.-Max. = Minimum - Maximum. < X = below limit of detection (LOD). CV = Coefficient of variation. n= number of observations; **Note:** Numbers of observations less than 10 in some districts (n = 4 districts) were due to exclusion of outliers.

**Table 3:** Age stratified Cd concentration (ng/g ww) in reindeer meat from the grazing districts with mixed age groups.

	District	
	Eastern Sør-Varanger	
Cd Concentration	Calves/Young (n = 8) (10 months–1.5 years)	Old (n = 2) (>2 years)
GM	0.74	1.5
AM	0.79	1.8
Min-Max	0.5–1.5	0.8–2.8
	**Varanger Peninsula**	
Cd Concentration	Calves/Young (n = 6) (10 months–1.5 years)	Old (n = 4) (>2 years)
GM	0.57	5.7
AM	0.58	6.3
Min-Max	0.4–0.7	2.8–10
	**Kandstadfjord/Western Hinnøy**	
Cd Concentration	Calves/Young (n = 6) (10 months–1.5 years)	Old (n = 4) (>2 years)
GM	3.4	5.2
AM	4.3	5.2
Min-Max	1.6–11	4.1–6.2

**Note:** The district Pasvik/Sør-Varanger: Calves/Young (n = 9): GM = 1.3, AM = 1.4, Min-Max: 0.8–2.1 ng/g ww; Old (n = 1): 7.6 ng/g ww.

### 3.3. Arsenic (As)

As was detected in all meat samples (100%). The As was the element that showed most of the geographical variations (Table 2) among the studied elements. An east-west downward geographical gradient was observed for As, with the highest concentrations measured in the east (the three districts in the vicinity of the Russian border). However, a north-south trend (highest in the south) for As, Cd and Pb has previously been found in Norwegian surface soils, coniferous forest ecosystems and some herbivorous animals [2,26,37,38,39]. 

Due to the wind frequency towards the districts of Eastern Sør-Varanger and Pasvik/Sør-Varanger, the high As concentration in these districts could be explained in this study by the As being released from the smelter activity in the Russian town of Nikel, which was further reported to be higher during summer as compared to winter [20]. Furthermore, the wind from the east (E) brings waste from the town of Nikel towards the direction of Svanvik/Passvik, while the wind from the north (N) and north-east (NE) brings waste from Zapoljarny during summer. The dominating wind in winter from the south (S) and south-west (SW) brings waste from Nikel to Jarfjord. However, the elevated As and Ni concentrations below the LOD in these two districts indicates additional As sources such as mining and geogenic sources in this area. The As concentrations in meat from Eastern Sør-Varanger (GM = 106.1 ng/g ww) and Pasvik/Sør-Varanger (GM = 47.9 ng/g ww) from this study were in agreement with those formerly revealed in liver samples from reindeer in the same area when compared with samples from other areas in the County [40]. By comparison, Bernhoft and colleagues reported median As concentration of 0.035 µg/g ww in reindeer liver collected from north western Russia [32].

The district of Ábborašša (gold mining activity) displayed As concentration (GM) three times higher than that found in the neighbouring district of Fávrrosorda, which could be explained by pollution from the mining work in the district, as As has been reported to be associated with gold mineralization [41,42]. In addition, the district of Spierttagáisá (military activity) revealed a lower As concentration (GM = 5.9 ng/g ww) than that (GM = 8.8 ng/g ww) found in the neighbouring district of Karasjok West, although the difference was not statistically significant. The As concentration in samples from Essand/Røros was similar to those detected in Ábborašša (gold mining activity) and Spierttagáisá (military activity). This could be due to the long-range atmospheric pollution from Europe, as a previous study has demonstrated that southern Norwegian areas are more prone to atmospheric pollution from Europe than the northern areas [43]. Contribution from soil due to geogenic sources could also lead to elevated As level in the surrounding environment. In accordance with previous study, the reindeer’s age and sex had no effect on the arsenic concentration [32].

### 3.4. Copper (Cu)

Cu was detected in all meat samples (100%) and had the highest concentration among all the studied elements (GM = 1,439 ng/g ww). Cu concentrations did not vary much among the districts (Table 2). Study on Finnish reindeer had also reported little differences in concentrations of Cu among different Lapland areas [15].

Cu concentrations detected in this study were in agreement with those reported from Finnish Lapland and Russian Karelia, and were half of those reported from Greenlandic reindeer muscle [15,17,33]. The results for the effect of age and sex on Cu concentrations from this study stand in contradiction to those reported by Bernhoft and colleagues, in which hepatic Cu concentration was higher in reindeer calves than in adult females, and higher in adult males than in adult females [32].

Districts that displayed relatively high Cu concentrations could be due to contamination from local point sources (gold mining in Ábborašša) and atmospheric transportation in Pasvik and Eastern Sør-Varanger (Russian towns of Nikel with its nickel smeltery and Zapoljarny with its briquette industry) [16]. 

### 3.5. Nickel (Ni)

Ni was detected in samples from five of the 10 districts (Fávrrosorda, Tromsdalen, Varanger Peninsula, Karasjok West and Essand/Røros) and in 20% of the total meat samples (n = 100). The Ni detection percentage within these five districts varied as follows: 80% (Fávrrosorda), 60% (Tromsdalen), 40% (Varanger Peninsula) and 10% in the districts of Karasjok West and Essand/Røros.

The districts of Varanger Peninsula, Fávrrosorda and Tromsdalen had geometric/arithmetic mean Ni concentrations above the LOD (Table 2), in which the Ni concentrations were comparable.

Previous studies on human Ni exposure along the Norwegian-Russian border have shown that urinary Ni concentrations in this area were no higher than the ones exhibited in other populations [44]. No data were available on Ni and V from reindeer muscle other than that of the Ni concentration from the Karelian Russian reindeer, which was reported to be below the LOD [33]. Nonetheless, the Ni concentrations formerly reported in liver and kidney samples from reindeer originating from the Norwegian-Russian border (Sør-Varanger, north eastern Norway and Rybatsjy Ostrov, north western Russia) had exhibited geographical variations and were much higher in levels than those documented in this study due to tissue differences [16,32]. 

### 3.6. Vanadium (V)

V was detected in 21% of the total meat samples (n = 100) and in seven of the 10 districts (Kanstadfjord, Tromsdalen, Pasvik, Spierttagáisá, Karasjok West, Eastern Sør-Varanger and Ábborašša). The detection percentages within these seven districts varied as follows: 50% (Spierttagáisá), 40% (Kanstadfjord), 30% (Eastern Sør-Varanger, Pasvik and Ábborašša) and 10% in Tromsdalen and Karasjok West. The district Spierttagáisá was the only one that had geometric/arithmetic mean V concentration (0.2/0.3 ng/g ww) above the LOD.

V has been described as a useful environmental pollution marker for the potential release of toxic elements from fossil fuels and oil refinery processes [45]. Results on V from Canadian (Yukon) caribou kidney reported an average concentration of 0.42 µg/g dry weight, 79.9% moisture [3].

### 3.7. Risk Assessment of Toxic Elements from Reindeer Meat Consumption

Reindeer meat is consumed as fresh, smoked or dried products in Norway, with the highest consumption among the indigenous Sami people, particularly reindeer herders and their families, compared to ethnic Norwegians [46]. The average consumption is generally low compared to other meat types and constitute approximately 23 g and 70 g/week for low and high consumers in areas with both Sami and ethnic Norwegians [47,48]. The estimated human toxic elements intake from reindeer meat based on the high consumption (70 g meat/week) were 0.01 and 0.01 µg/kg human body weight for Cd and As, respectively. These estimations (monthly for Cd and weekly for As) were much lower than permissible tolerable monthly intake (PTMI) for Cd (25 µg/kg human body weight) and weekly intake (PTWI) of 15 µg/kg human body weight for As [49,50]. The FAO/WHO-JECFA has recently withdrawn the PTWI limit of 25 µg/kg human body weight for Pb due to its association with a decrease of at least three intelligence quotient (IQ) points in children and an increase in systolic blood pressure of approximately 3 mmHg in adults [49]. No new PTWI limit was established for Pb. Nevertheless, weekly human Pb exposure from reindeer meat of 0.002 µg/kg human body weight was 12,500 times lower than the previous PTWI limit. The estimated daily Cu intake from reindeer meat in this study was about 0.0003 mg/kg body weight which was well below the acceptable daily intake (ADI) of 0.5 mg/kg body weight [51]. There are no established PTWI/PTMI limits for Ni and V. Nevertheless, concentrations of Ni and V detected in the present study were considerably lower than tolerable upper intake levels of 1 mg and 1.8 mg per day that have been reported elsewhere for Ni and V, respectively [52].

Estimation of human exposure to toxic elements through reindeer meat was previously done based on dietary data from a questionnaire on the Population-based Health and Living Conditions in areas with Sami and Norwegian populations–The SAMINOR Study and the equation described in one of our previous studies [47,48]. The dietary data from the questionnaire and calculations based on the equation mentioned above have revealed considerably low human exposure to toxic elements through meat and other edible tissues from reindeer.

The presence of individual animals with elevated toxic element concentrations (outliers) was investigated further by relating these concentrations to concepts such as maximum levels (ML), acceptable daily intakes (ADI), professional tolerable intakes (PTI) and healthy animals’ parameters [12,49,50,53]. For instance, the elevated concentrations of outliers in cases of cadmium (13 and 15 ng/g ww) and lead (23 and 28 ng/g ww) have been estimated to constitute 20% and 40% of the maximum levels (ML) set for Cd, and 20% and 30% of the ML set for Pb [53]. Consequently, the elevated concentrations measured in this study should not be an issue of concern to consumers.

## 4. Conclusions

Arsenic and cadmium were the elements that exhibited most of the geographical differences. No clear geographical trend was observed except for the east-west gradient for As, with the highest concentrations measured in the east (near the Russian border). The presence of older animals (>2 years) displayed an age effect as animals more than 2 years old demonstrated higher cadmium concentration than ones <2 years old, whereas sex had no significant effect on toxic element concentrations. The concentrations of the toxic elements detected in this study were low and considerably below the maximum levels (ML) and permissible tolerable weekly/monthly intake (PTWI/PTMI) limits available for hazardous toxic elements. This suggests that the use of reindeer meat as human food is safe in relation to toxic elements, even along the Norwegian-Russian border where previous studies have revealed elevated concentrations in liver and kidneys from reindeer [16]. Based on the result from the present study, we have no reason to warn people against eating reindeer meat. Further investigations regarding arsenic findings are needed. 

## Supplemental Material

**Table S1:** Geographical differences in concentrations of the studied toxic elements among the ten grazing districts.

Toxic element	n	District X: District Y (P-value)			
Cd	98				
		**Kanstadfjord:** Tromsdalen **	**Kanstadfjord:** Spierttagáisá **	**Kanstadfjord:** Karasjok **	**Kanstadfjord:** E. S-Varanger **
		Tromsdalen: **Fávrrosorda** **	Tromsdalen: **Essand** **	**Fávrrosorda:** Varanger P **	**Fávrrosorda:** Pasvik *
		**Fávrrosorda:** Spierttagáisá **	**Fávrrosorda:** Karasjok **	**Fávrrosorda:** E. S-Varanger **	Spierttagáisá: **Essand** **
		Karasjok: **Essand****	E. S-Varanger: **Essand** **		
Pb	98				
		Tromsdalen: **Fávrrosorda** **	**Fávrrosorda:** Spierttagáisá *	**Fávrrosorda:** Karasjok **	**Fávrrosorda:**E. S-Varanger **
		**Fávrrosorda:** Ábborašša **	**Pasvik:** E. S-Varanger *	E. S-Varanger: **Essand** **	
As	99				
		**Kanstadfjord:** Tromsdalen **	**Kanstadfjord:** Fávrrosorda **	Kanstadfjord: **Varanger P** **	Kanstadfjord: **Pasvik** **
		Kanstadfjord: Karasjok **	Kanstadfjord:**E. S-Varanger** **	Kanstadfjord: **Essand** *	Kanstadfjord: **Ábborašša** *
		Tromsdalen: **Varanger P** **	Tromsdalen: **Pasvik** **	Tromsdalen: **Spierttagáisá ****	Tromsdalen: **Karasjok** **
		Tromsdalen: **E. S-Varanger** **	Tromsdalen: **Essand** **	Tromsdalen: **Ábborašša ****	Fávrrosorda: **Varanger P** **
		Fávrrosorda: **Pasvik** **	Fávrrosorda: **Spierttagáisá** **	Fávrrosorda: **Karasjok** **	Fávrrosorda:**E. S-Varanger** **
		Fávrrosorda: **Essand** **	Fávrrosorda: **Ábborašša** **	Varanger P: **Pasvik** **	**Varanger P:** Spierttagáisá *
		Varanger P:**E. S-Varanger** **	**Pasvik:** Spierttagáisá **	**Pasvik:** Karasjok **	Pasvik: **E. S-Varanger** **
		**Pasvik:** Essand **	**Pasvik:** Ábborašša **	Spierttagáisá: **E. S-Varanger** **	Karasjok: **E. S-Varanger** **
		**E. S-Varanger:** Essand**	**E. S-Varanger:** Ábborašša**		
Cu	98				
		Kanstadfjord: **Pasvik** *	Kanstadfjord: **E. S-Varanger** *	Fávrrosorda: **Pasvik** **	Fávrrosorda: **E. S-Varanger** **
		Fávrrosorda: **Essand** *	Fávrrosorda: **Ábborašša** *		

**Legend:** n = number of observations. Level of significance *: 0.01 < *p* < 0.05, **: *p* < 0.01. **Note:** Districts in **bold** indicate the highest concentrations.
